# The effects of supported employment interventions in populations of people with conditions other than severe mental health: a systematic review

**DOI:** 10.1017/S1463423621000827

**Published:** 2021-12-09

**Authors:** Katrin Probyn, Martin Stav Engedahl, Dévan Rajendran, Tamar Pincus, Khadija Naeem, Dipesh Mistry, Martin Underwood, Robert Froud

**Affiliations:** 1 Department of Psychology, Royal Holloway, University of London, Egham Hill, Egham, Surrey, UK; 2 Department of Health Sciences, Kristiana University College, Oslo, Norway; 3 European School of Osteopathy, Boxley, Maidstone, Kent, UK; 4 Warwick Clinical Trials Unit, Warwick Medical School, University of Warwick, Coventry, UK; 5 University Hospitals Coventry & Warwickshire, University of Warwick, Coventry, UK

**Keywords:** individual placement and support, IPS, occupational rehabilitation, return to work, supported employment, TVR, vocational rehabilitation

## Abstract

**Aim:**

To assess the effectiveness of supported employment interventions for improving competitive employment in populations of people with conditions other than only severe mental illness.

**Background:**

Supported employment interventions have been extensively tested in severe mental illness populations. These approaches may be beneficial outside of these populations.

**Methods:**

We searched PubMed, Embase, CINAHL, PsycInfo, Web of Science, Scopus, JSTOR, PEDro, OTSeeker, and NIOSHTIC for trials including unemployed people with any condition and including severe mental illness if combined with other co-morbidities or other specific circumstances (e.g., homelessness). We excluded trials where inclusion was based on severe mental illness alone. Two reviewers independently assessed risk of bias (RoB v2.0) and four reviewers extracted data. We assessed rates of competitive employment as compared to traditional vocational rehabilitation or waiting list/services as usual.

**Findings:**

Ten randomised controlled trials (913 participants) were included. Supported employment was more effective than control interventions for improving competitive employment in seven trials: in people with affective disorders [risk ratio (RR) 10.61 (1.49, 75.38)]; mental disorders and justice involvement [RR 4.44 (1.36,14.46)]; veterans with posttraumatic stress disorder (PTSD) [RR 2.73 (1.64, 4.54)]; formerly incarcerated veterans [RR 2.17 (1.09, 4.33)]; people receiving methadone treatment [RR 11.5 (1.62, 81.8)]; veterans with spinal cord injury at 12 months [RR 2.46 (1.16, 5.22)] and at 24 months [RR 2.81 (1.98, 7.37)]; and young people not in employment, education, or training [RR 5.90 (1.91–18.19)]. Three trials did not show significant benefits from supported employment: populations of workers with musculoskeletal injuries [RR 1.38 (1.00, 1.89)]; substance abuse [RR 1.85 (0.65, 5.41)]; and formerly homeless people with mental illness [RR 1.55 (0.76, 3.15)]. Supported employment interventions may be beneficial to people from more diverse populations than those with severe mental illness alone. Defining competitive employment and increasing (and standardising) measurement of non-vocational outcomes may help to improve research in this area.

## Background

Conventional traditional vocational rehabilitation (TVR) tends to follow a train-and-place model; often involving pre-employment training, testing, or counselling to prepare individuals for employment, and often involving sheltered employment (Bond *et al*., 2012). Supported employment interventions emphasise a place-and-train approach and placement of individuals (who have health problems but would like to work) in real-world work settings, then providing the support that is needed (Bond, 2004). Individual placement and support (IPS) is a standardised model of supported employment that has been developed for people with severe mental illness, which is defined as schizophrenia or schizophrenia-like disorder, bipolar disorder, or major depression with psychotic features (Drake *et al.*, 1999; Crowther *et al.*, 2001; Kinoshita *et al*., 2013).

IPS was developed in the USA in the 1990s. It is based on eight principles and aims to secure competitive employment. The focus of the intervention is on providing preference-based supported employment for those who want to work, integration of services, the provision of benefits counselling, a rapid job search, and ‘time-unlimited’ support (Drake *et al.*, 1999; Drake, 2012b; Becker *et al.*, 2019). Supported employment more generally operates around the central concept that people who want to work can be placed in a job they want and then receive appropriate support. IPS and supported employment in populations involving severe mental illness alone have been extensively evaluated (Kinoshita *et al*., 2013; Crowther *et al*., 2001; Modini *et al.*, 2016; Marshall *et al*., 2014; Suijkerbuijk *et al.*, 2017). This individualised approach is gaining support and beginning to be implemented in new populations other than those with severe mental health conditions (Drake, 2012a; 2012b). However, it is not clear how effective such interventions are in these populations. Our aims were to assess the effectiveness of any type of supported employment intervention (including IPS) for improving rates of competitive employment in populations of people for whom severe mental illness is not their problem or not their only problem; to describe and report secondary outcomes measured; to summarise definitions of competitive employment used; and to extract summary details of tested interventions.

## Methods

We prospectively registered the systematic review with the International Prospective Register of Systematic Reviews: PROSPERO (NIHR, 2017) and followed the PRISMA (Preferred Reporting Items for Systematic Reviews and Meta-Analyses) guidelines for reporting of systematic reviews (Moher, 2009). The review was part of a larger study (Return to work with Individualised Supported Employment – RISE), which was funded by Versus Arthritis, and was designed to determine the feasibility of delivering an individualised supported work placement intervention to people with chronic pain. The early phases of the present review informed the intervention development process and the choice of outcome measures used in the RISE intervention (Froud *et al.*, 2020).

### Search

We searched for peer-reviewed randomised controlled trial (RCT) reports in PubMed, Embase, CINAHL, PsycInfo, Web of Science, Scopus, JSTOR, PEDro, OTSeeker, and NIOSHTIC from database inception to March, 2020. We based our search on MeSH indexing terms and free text terms relating to supported employment intervention. An example search string is included in Appendix 1. We supplemented our search with backwards citation tracking. Only trials published in English were included. We excluded grey literature and conference proceedings.

### Eligibility criteria

Papers reporting RCTs were eligible for inclusion if they met the following criteria:

(1) They assessed effectiveness of a supported employment intervention compared to TVR waiting list/services as usual; (2) The intervention aim was to obtain and maintain competitive employment; (3) Study participants were unemployed at the beginning of the trial; and (4) Rates of obtaining competitive employment were measured and reported as an outcome.

Papers describing trials of supported employment intervention for participants with any problem other than severe mental health illness alone were included. Pilot studies were included if they reported smaller scale RCTs and included an objective of estimating effectiveness parameters. Conversely, feasibility studies that did not conduct a small-scale RCT and estimate effectiveness were excluded. For those with severe mental illness, we included RCTs where an additional co-morbidity or a materially different population was involved compared to severe mental illness alone; for example, severe mental illness and homelessness. Studies were excluded if they were not RCTs, not published in a peer-reviewed journal, or the target population did not comprise adults of working age.

### Study selection process and data extraction

The search results were managed using EPPI reviewer 4 software (*EPPI-Centre at the Social Science Research Unit of the UCL Institute of Education, University of London, London, UK)*. Two of four reviewers (KP, KN, MSE, or DR) independently screened all records by title and abstract. Disagreements were resolved through discussion and, if necessary, with a third reviewer acting as an arbitrator. Articles that could not be excluded from titles and abstracts sifting were retrieved as full text and assessed independently against inclusion criteria. Two reviewers independently extracted data. For each study, data on the country of the trial, intervention and control arm, structure and delivery of interventions, fidelity to any relevant instrument [e.g., an edition of the Supported Employment Fidelity Manual (Becker *et al.*, 2008)], competitive employment rates, definitions of competitive employment used, secondary vocational and non-vocational outcomes, and length of follow-up time were abstracted.

### Appraisal of quality

Two reviewers (MSE, DR) independently assessed the risk of bias using the Cochrane Risk of Bias Tool v2.0 (Sterne *et al*., 2019). Disagreements were resolved through discussion with a third acting as arbitrator if necessary.

### Data synthesis

Our *a priori* primary outcome (PROSPERO 2017:CRD42017067586) for all comparisons of effectiveness was ‘obtaining competitive employment’. We extracted numbers returning to work in intervention and control groups. We abstracted author-reported risk ratios (RRs) and 95% confidence intervals, or calculated these from reported rates in cases where RRs were not reported by authors. We planned a meta-synthesis, and a meta-regression of study characteristics on effects, in the case of sufficient homogeneity – either overall, or within strata of sub-populations. In the case of high population heterogeneity and the absence of any such strata, we did not meta-analyse and presented obtaining competitive employment RRs from studies, making a forest plot for illustrative purposes only, and without estimating any pooled effects. We note that random effects models assume that underlying effects follow a normal distribution (Higgins *et al*., 2021). This assumption is not credible in case included studies span very different populations. *P*-values for between-group differences were extracted as reported by original authors, and authors’ descriptive statistics were used to summarise secondary outcomes of included studies. Assessment of publication bias was determined through visual inspection of funnel plots. All analyses were done using Stata Version 15.1 (IBM, Washington).

## Results

**Figure 1:**
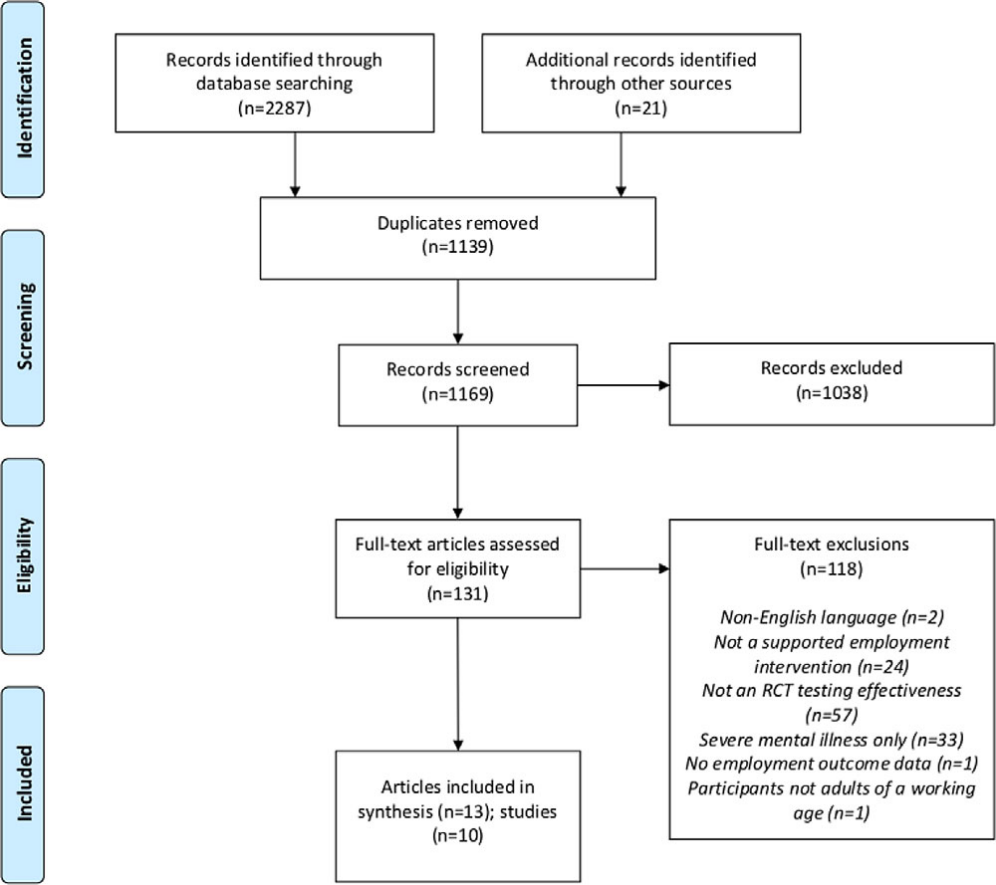
Flow chart showing details of records identified, screened, assessed for eligibility, and included in the review.

We included 13 articles describing 10 trials. Figure 1 shows a flow chart of the search process and included articles (Figure 1, Flow chart).

The 10 included trials (913 participants) used supported employment interventions across different populations (Table 1) (Bejerholm *et al*., 2017; Bond *et al.*, 2015; Davis *et al.*, 2012; LePage *et al*., 2016; Li-Tsang *et al*., 2008; Magura *et al.*, 2007; Ottomanelli *et al*., 2012; Poremski *et al*., 2015; Lones *et  al.*, 2017; Sveinsdottir *et al*., 2020). Each compared to some form of TVR/services as usual, as per inclusion criteria. One paper reporting follow-up data for one of the included trials and two available protocols for included trials were also included in our review material (Ottomanelli *et al*., 2014; Ottomanelli *et al*., 2009; Sveinsdottir *et al.*, 2016).

**Table 1:** Characteristics of included studies

Lead author, year, *n* randomised, Country	Population	Intervention	Control	Follow-up (months)	Compliance^*^/attrition	RTW Int.	RTW contr.
Bejerholm, 2017, *n =* 63, Sweden	Affective disorders	‘Individual Enabling and Support (IES) program’	TVR	12	92%; Drop-outs; IPS: ineligible after randomisation (*n* = 2); control: loss to follow-up (*n* = 3)	42%	4%
Bond, 2015, *n =* 90, USA	Severe mental illness+ justice involvement	IPS with special legal training/information for case manager	Work readiness classes	12	94.4%; Drop-outs; ineligible post-randomisation (*n* = 1); overruled consent (*n* = 1); offered incorrect intervention (*n* = 1); missing data (*n* = 2); not reported by randomised group	31%	7%
Davis, 2012, *n =* 85, USA	Veterans with PTSD	IPS incorporated with PTSD clinical treatment team	‘Veterans’ Health Administration Vocational Rehabilitation Program (VRP)’	12	84%; IPS group: Withdrawn consent (*n* = 1), relocation (*n* = 3), and incarceration (*n* = 2); VRP group: Lost to follow-up (*n* = 2), relocation (*n* = 5), and incarceration (*n* = 1)	76%	28%
Le Page, 2016, *n =* 88 USA	Formerly incarcerated veterans with formal diagnosis of a substance-use disorder, mental illness, or both	Based on IPS, although it is noted that the intervention is not classified as IPS and is not classed as IPS in terms of fidelity. incorporated in existing vocational programme for veterans (AF)	About Face program (AF): 1-week standardised vocational rehabilitation group based programme	6	95.4%; Drop-outs; found employment prior to allocation (*n* = 2); not medically cleared for employment (*n* = 2)	46%	21%
Li-Tsang, 2008*, n =* 63, Hong Kong	Workers with musculoskeletal injuries	3-week job replacement programme with case management approach with goal of rapid placement	Self-placement group – given advice on job placement at a workers’ health centre	0.75	95.2%; Drop-outs; intervention group (personal reasons (*n* = 2)) control group (personal reasons (*n* = 1))	84%	61%
Lones, 2017, *n =* 45, USA	Moderate to severe opioid use disorder, receiving methadone treatment	IPS conducted in the treatment site’s clinic and outside the clinic setting	IPS waiting list + services as usual	12	77%; Lost to follow-up (*n* = 10); six in IPS group and four in waitlist group): incarcerated (*n* = 2), withdrew consent (*n* = 1), unknown (*n* = 7)	50%	4%
Magura, 2007, *n =* 213, USA	Substance-misuse methadone patients	Customised employment and support model; case-management approach with goal of rapid placement	Standard vocational counselling	12	78.8%; Drop-outs (could not be located; *n* = 16); ineligible post randomisation (*n* = 2); did not receive counselling (*n* = 24); group allocations not reported	10%	6%
Ottomanelli, 2012, 2014, *n =* 157, USA	Veterans with spinal cord injury	IPS incorporated with spinal cord injury rehabilitation team at Veterans Affairs centre	Treatment-as-usual (TAU) involving referrals to VR services outside the Veterans Affairs (VA) SCI centre	12, 24	From 2012 paper; 12m: 86.6% Drop-outs in randomised groups; SE (*n* = 14) TAU (*n* = 7) Reasons not provided by randomised groups. From 2014 paper (12m): 89.1%. Drop-outs in randomised groups; SE (*n* = 10) TAU (*n* = 7) Reasons not provided. 24m: 51.2% (from baseline). Drop-outs in randomised groups; SE (*n* = 38) TAU (*n* = 34) Reasons not provided	26% (Year 1) 19% (Year 2)	11% (Year 1) 7% (Year 2)
Poremski, 2015, *n =* 90, Canada	Mental illness + homeless and recently housed	IPS	Non-integrated employment services (TAU)	12	94.4% Drop-outs; IPS: loss to follow-up (*n* = 1) TAU: loss to follow-up (*n* = 1); death (*n* = 3)	34%	22%
Sveinsdottir, 2020, *n* = 96 Norway	Young people not in employment, education, or training	IPS	TVR	12	86.4%; Loss to follow-up (*n* = 13); 4 in IPS group and 9 in TVR group	48%	8%

AF, about face programme; IES, individual enabling and support program; IPS, individual placement and support; PTSD, posttraumatic stress disorder; SCI, spinal cord injury; SMI, severe mental illness; SE, supported employment; SP, self-placement group; SS, standard services; RTW, return to work; TAU, treatment as usual; TVR = traditional vocational rehabilitation; VRP, veterans health administration vocational rehabilitation program; VA, veterans affairs; int. = intervention, contr. = control.

* Percentage of participants retained in the study at follow-up.

Table 1 shows characteristics of the included trials. We judged that six trials used IPS as a supported employment intervention and authors of these trials reported assessing fidelity against a scale (Appendix Table 1) (Becker *et al*., 2001; Becker *et al*., 2015; Becker *et al*., 2008). In four trials, we judged that other supported employment interventions were tested (Appendix Table 1).

Two trials included participants with physical disabilities: one studied supported employment for veterans with spinal cord injury (Ottomanelli *et al*., 2009; Ottomanelli *et al.*, 2012; Ottomanelli *et al*., 2014) and one for workers with musculoskeletal injuries (Li-Tsang *et al*., 2008). Two trials included substance-misuse (methadone) patients (Magura *et al*., 2007; Lones *et al.*, 2017). In inclusion criteria, Li-Tsang *et al.* describe that participants must have been injured or on sick-leave for at least 6 months. However, participants are referred to as being unemployed in the Discussion section, and it is clear from Li-Tsang’s description of methods that participants are being helped to find new jobs. The study was based in Hong Kong, where under the Employment Ordinance employees can only accrue employer-paid sickness for up to 120 days, for example <6 months (HK Labour Department, 2021). Thus, we reasoned that participants cannot have been in paid employment at the point of recruitment and judged that the trial met the inclusion criteria of this review. Lones *et al*. is a pilot study that featured a small-scale RCT and included as an objective in the estimation of effectiveness of the intervention on employment (Lones *et al.*, 2017). Bejerholm *et al.* (2017) included people with less debilitating mental illness and participants with affective disorders. Bond included people with severe mental illness alongside justice involvement (Bond *et al*., 2015). Two trials included veterans: one veterans with PTSD; (Davis *et al.*, 2012) and one formerly incarcerated veterans with substance-use disorder and/or mental illness (LePage *et al*., 2016). Magura *et al*. (2007) included substance-misuse methadone patients. Poremski *et al*. (2015) included people with mental illness and who were previously homeless but had been recently housed. Finally, Sveinsdottir *et al*. (2020) included young adults with various social or health-related problems who were at risk of work disability. The reported caseloads across all included studies, per employment specialist, ranged from 15 to 35.

### Risk of bias

**Figure 2:**
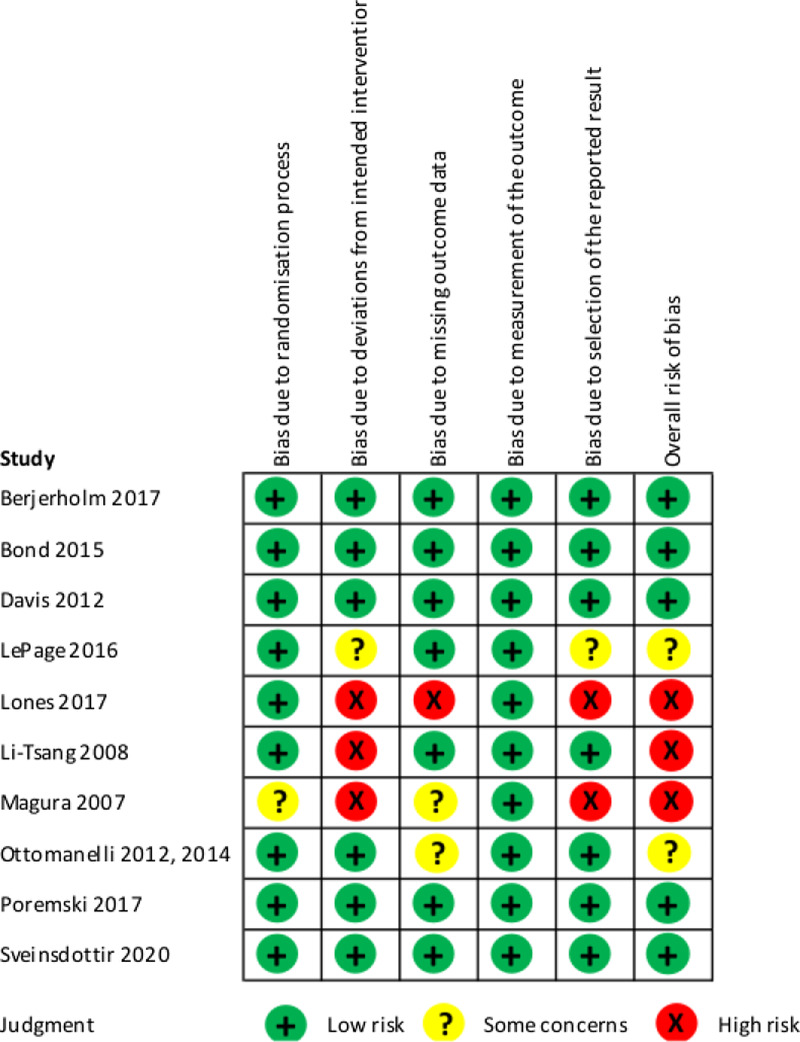
Risk of Bias assessment of included studies.

We judged five studies (5/10; 50%) to have an overall ‘low risk’ of bias, which reflected the scores of ‘low risk’ across all five domains. Three (3/10; 30%) were judged to have an overall ‘high risk’ of bias, which reflected judgements of high risk in at least one of the five domains. Two (2/10; 20%) were judged to have ‘some concerns’, reflecting judgements of some concerns in at least one of the five domains in the absence of judgements of high risk in these domains (Figure 2).

### Obtaining competitive employment

All of the included trials assessed obtaining competitive employment as a primary outcome. The phrase ‘competitive employment’ was either elaborated on or more explicitly defined in eight of the trials (Appendix Table 2). Three of the trials gave details of how long a job must be held to qualify as meeting the criterion of competitive employment: two stated for a minimum of one day, and one for a period of four weeks for a minimum of 18 hours/week.

**Figure 3:**
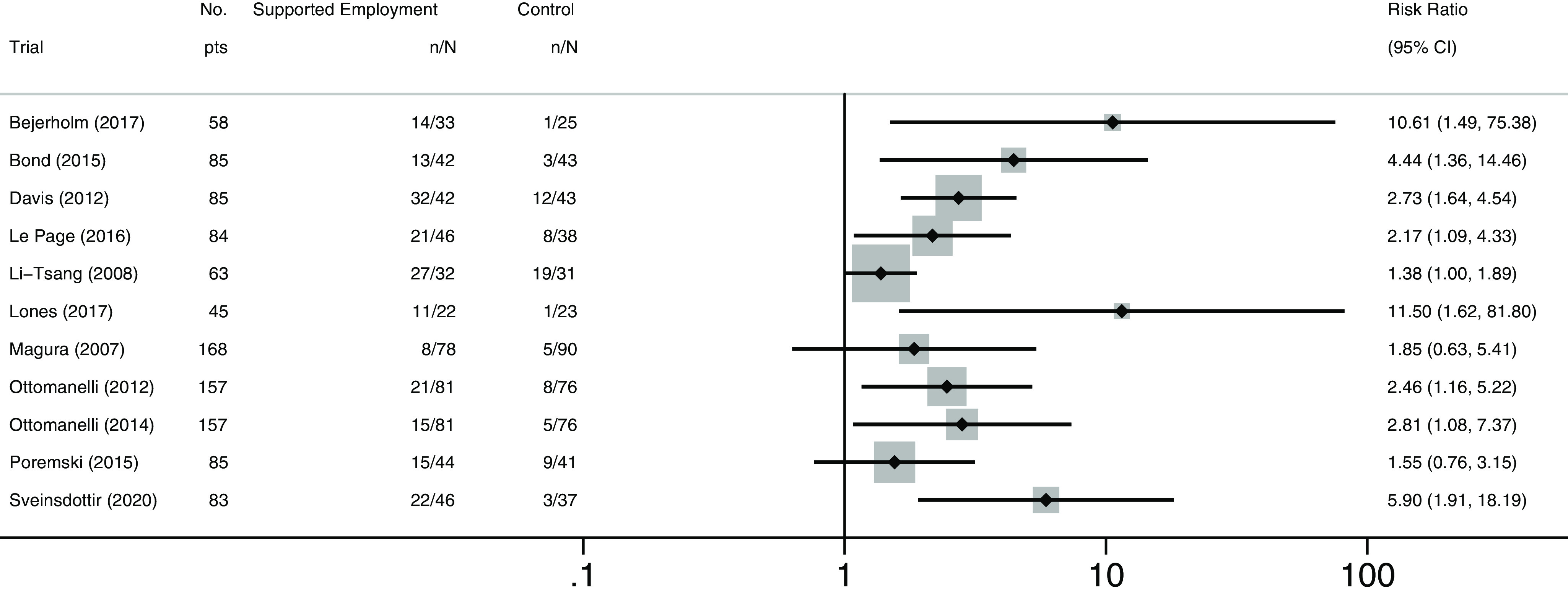
Forest plot of risk ratios for obtaining competitive employment, by study (cf. Table 1 for population details. *n* reflects numbers analysed and may differ from number randomised).

**Figure 4:**
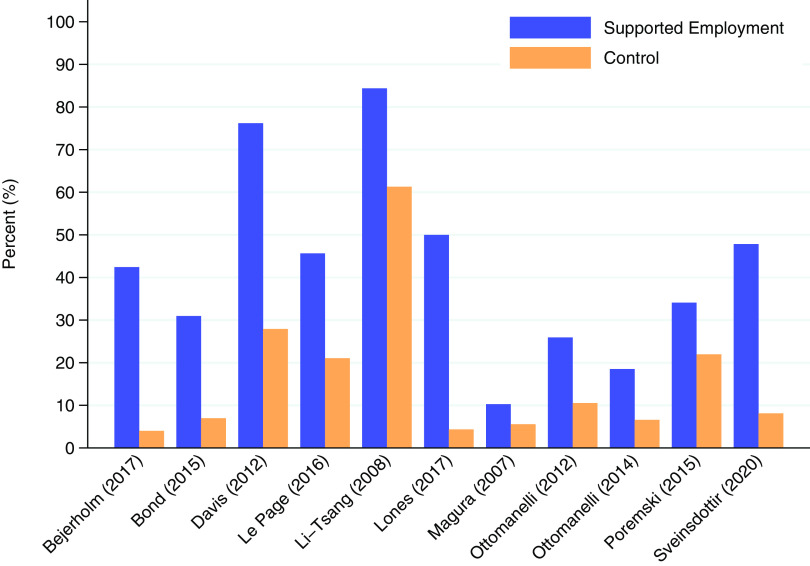
Participants obtaining competitive employment (cf. Table 1 for population details).

Figure 3, which is a forest plot for illustrative purposes only, shows that in 8 of the 11 (80%) included trial result rows (*n.b.* where one row is a two-year follow-up of the same trial), supported employment was more effective than control in returning people to work. Supported employment was more effective than TVR in people with affective disorders, where the outcome was competitive employment at 12 months [RR 10.61 (95% CI 1.49 to 75.38)] (Bejerholm *et al*., 2017); in people with severe mental illness and justice involvement where the outcome was competitive employment at 12 months (RR 4.44 (1.36 to 14.46)) (Bond *et al*., 2015); in veterans with posttraumatic stress disorder (PTSD) where the outcome was competitive employment within 12 months [RR 2.73 (1.64 to 4.54)] (Davis *et al.*, 2012); in formerly incarcerated veterans, where the outcome was competitive employment at 6 months [RR 2.17 (1.09 to 4.33)] (LePage *et al*., 2016); in people receiving methadone treatment, where the outcome was competitive employment at 6 months [RR 11.5 (1.62 to 81.80)] (Lones *et al*., 2017); in veterans with spinal cord injury, where the outcome was competitive employment within 12 months [RR 2.46 (1.16 to 5.22)] and within 24 months [RR 2.81 (1.98 to 7.37)] (Ottomanelli *et al*., 2012; Ottomanelli *et al*., 2014); and in young people not in employment, education, or training, where the outcome was any (expressed as weeks, days, or hours) competitive employment (minimum of 1 day) at 12 months [RR 5.90 (1.91 to 18.19)] (Sveinsdottir *et al*., 2020). Supported employment interventions were not shown to be more effective than control in returning people to work in three trials: one trial including workers with musculoskeletal injuries, where the outcome was continuous employment for four weeks or more for at least 18 hours/week and within the 3-week period following the programme (*sic* ^†^) [RR 1.38 (1.00 to 1.89)] (Li-Tsang *et al*., 2008); one trial including substance-misuse methadone patients, where the outcomes was competitive employment within 12 months [RR 1.85 (0.65 to 5.41)] (Magura *et al*., 2007); and one trial including homeless people with mental illness who were recently housed, where the outcome was competitive employment of at least one day within a 30-day period [RR 1.55 (0.76 to 3.15)] (Poremski *et al.*, 2015). As heterogeneity was high across the different included populations, and the assumption of an underlying normal distribution of treatment effects not plausible, we did not do a meta-analysis. We note that the two papers by Ottomanelli, reporting 12 and 24 month results on the same participants, are both included in Figures 3 and 4 (since we did not meta-analyse). Figure 4 shows the comparison of percentages of participants returned to work in supported employment versus their corresponding control groups.

### Other vocational outcomes

Table 2 summarises other vocational outcomes reported in the included studies. Not every domain in the table was reported in each study. Total hours worked were significantly greater than control for those receiving supported employment in three of the four studies reporting on this outcome (formerly incarcerated veterans; veterans with spinal cord injuries, and young people not in employment, education, or training). Hours worked per week significantly favoured supported employment in four of the six reporting studies (People with affective disorders; formerly incarcerated veterans; veterans with spinal cord injuries; and young people not in employment, education, or training). Four of seven studies, that included a measure on wages/income earned reported a significant difference in income earned when receiving supported employment (affective disorders; veterans with PTSD; formerly incarcerated veterans; and veterans with spinal cord injury in the second year). Two studies assessed mean number of weeks worked during study period, reporting these as significantly favouring supported employment (affective disorders and veterans with PTSD). Two studies assessed the mean days worked during study period with both reporting a significant effect favouring supported employment (people with severe mental illness and justice involvement; and veterans with PTSD). Finally, one study reported obtaining any paid employment and any informal paid employment as an outcome in addition to obtaining competitive employment and reported significant effects in both cases for those receiving supported employment.

**Table 2:** Additional vocational outcomes

Study ID, population	Outcome measure and follow-up time	*n* treatment	Mean or (%)	SD	*n* control	Mean or (%)	SD	*P*-value^*^
**Work hours (total)**								
Bejerholm, 2017, Affective disorders	Employment hours at 12-month follow-up	33	210.39	432.8	25	3.84	19.2	0.01
Davis, 2012, Veterans with PTSD	Hours competitively employed at 1 year	42	656	661	43	236	494	<0.001
Le Page, 2016, Veterans with felony history, substance use disorder, mental illness, or both	Total hours worked over 6-month follow-up period	46	130.1	222.7	38	52.3	130.6	0.03
Sveinsdottir, 2020, Young people not in employment, education, or training	Total number of hours worked over 12-month follow-up	43	140.02	249.4	37	13.95	55.48	0.002
**Hours worked per week**								
Bejerholm, 2017, Affective disorders	Hours per week at 12-month follow-up	33	10.97	17.28	25	0.32	1.6	0.003
Le Page, 2016, Veterans with felony history, substance use disorder, mental illness, or both	Hours worked per week during 6 months	46	17.4	25.6	38	7.9	17.4	0.04
Li-Tsang, 2008, Musculoskeletal injuries	Mean working hours per week; 3-week follow-up	32	33.9	20.6	31	32.2	20.2	0.79
Ottomanelli, 2012, Veterans with Spinal cord injury	Hours worked per week during 1 year among all subjects (ITT)	81	6.5	1.5	76	2.0	1.6	<0.001
	Hours worked per week during 2 years among all subjects (ITT)	81	4.1	7.9	76	1.7	6	<0.001
Poremski, 2015, SMI+ homeless; recently rehoused	Hours per week in competitive work during jobs at 12 months	44	26.0	ND	41	25.8	ND	0.98
Sveinsdottir, 2020, Young people not in employment, education, or training.	Percentage of participants ever-working >/= 20 h/week over 12-months follow-up	42	(33.3%)	–	37	(5.41%)	–	0.002
**Wages/income earned**								
Bejerholm, 2017, Affective disorders	Net income (Euro) at 12 months	54 (both groups)	1565	ND	54 (both groups)	1048	ND	<0.001
Davis, 2012, Veterans with PTSD	Total gross 12-month income (mean): competitive sources ($)	42	9,264	13 294	43	2601	6009	<0.001
Li-Tsang, 2008, Musculoskeletal injuries	Mean monthly income during 3 weeks (HK$)	32	4,468	3,145	31	2,958	2,434	0.07
Magura, 2007, Substance abuse methadone patients	Income from any paid employment during study period ($), at 12-month follow-up	78	3707	7221	90	3914	7119	NS
Ottomanelli, 2012, 2014 Veterans with Spinal cord injury	Wages per week ($) during Year 1 among all participants (ITT)	81	69.3	209.3	76	31.7	325.1	0.39
	Wages per week ($) during Year 2 among all participants (ITT)	81	52.7	102.8	76	5.6	19.1.	<0.001
Le Page, 2016, Veterans with felony history, substance use disorder, mental illness, or both	Total wages ($) during 6-month follow-up	46	1401	2477	38	694	1749	0.04
Poremski, 2015, SMI+ homeless (recently rehoused)	Wage/hour ($) for competitive work at 12 months	44	13.84	ND	41	12.81	ND	0.42
**Weeks worked**								
Bejerholm, 2017, Affective disorders	Weeks worked in employment at 12-month follow-up	33	7.73	13.41	25	0.64	2.5	0.005
Davis, 2012, Veterans with PTSD	Weeks competitively employed at 1 year	42	21.6	17.7	43	6.8	13.8	<0.001
**Days worked**								
Bond, 2015, SMI and Justice involvement	Mean days of competitive employment during 1-year follow-up	42	40.5	99.2	43	15.9	65.7	<0.01
Davis, 2012, Veterans with PTSD	Days competitively employed at 1 year	42	83.8	80.6	43	29.3	61.9	<0.001
**Tenure of competitive employment**								
Poremski, 2015, SMI+ homeless (recently rehoused)	Days, for those who obtained competitive jobs during the study. 12-months follow-up	44	116.8	ND	41	102.9	ND	0.99
**Number of jobs**								
Li-Tsang, 2008, Musculoskeletal injuries	Number of jobs taken during 3-week intervention	32	1.49	0.8	31	1.08	0.29	0.31
**Paid employment**								
Magura, 2007, Substance abuse methadone patients	Any paid employment	78	(41%)	–	90	(26%)	–	<0.05
	Informal paid employment	78	(27%)	–	90	(14%)	–	<0.05

ITT, intention to treat population; ND, no data; PTSD, posttraumatic stress disorder; SMI, severe mental illness.

* *P*-value as reported verbatim as per study reports (*e.g.,* NS/*P*</*P*=).

### Non-vocational outcomes

Table 3 summarises non-vocational outcomes reported in the included studies. Again, not every domain in the table was reported in each study. Satisfaction with employment services was significantly greater than control for those receiving supported employment in a population of homeless people with mental illness. Quality of life was significantly improved over control for those receiving supported employment in workers with musculoskeletal injuries, and stress and anxiety was significantly lower in the same population group.

**Table 3:** Non-vocational outcomes

Study ID Population	Follow-up time (and measure)	*n* treatment	Mean (%)	SD	*n* control	Mean (%)	SD	*P*-value^*^
Psychiatric hospital admissions During 1-year follow-up								
Bond, 2015, SMI and Justice involvement	Mean number of hospitalisations	41	1.20	1.58	43	0.70	1.04	NS
	Mean days hospitalised	41	10.44	23.07	43	4.93	7.59	NS
Involvement with the criminal justice system: obtained participant self-report of arrests, convictions, and incarcerations during 1-year follow-up								
Bond, 2015, SMI and Justice involvement	Arrests	41	(24%)	–	43	(19%)	–	NS
	Felony convictions	41	(0%)	–	43	(0%)	–	NS
	Misdemeanour convictions	41	(2%)	–	43	(2%)	–	NS
	Incarcerations	41	(2%)	–	43	(2%)	–	NS
Self-reported recovery								
Bond, 2015, SMI and Justice involvement	Recovery Assessment Scale at 12 months 24-item subscale of the 32-item Recovery Assessment Scale	42	4.14	0.57	43	4.14	0.49	NS
Satisfaction with employment services								
Poremski, 2015, SMI+ homeless (recently rehoused)	Satisfaction with the vocational services received at 12-month follow-up	33	41	8	22	34	12	<0.001
Stress and anxiety levels								
Li-Tsang, 2008, Musculoskeletal injuries	State Trait and Anxiety Inventory (C-STAI), post-interventions	32	49.71	10.08	31	58.23	8.32	0.03
Sveinsdottir, 2020, Young people not in employment, education, or training	HSCL (Hopkins Symptom Checklist: total) (6 months)	38	1.74	0.59	24	1.95	0.54	0.165
	HSCL total (12 months)	30	1.79	0.63	26	2	0.6	0.22
	TOMCATS coping (Theoretically originated measure of the cognitive activation theory of stress: coping 6 months	39	2.77	0.78	23	2.83	0.49	0.725
	TOMCATS coping 12 months	29	2.79	0.62	25	2.76	0.88	0.872
	TOMCATS Helplessness 6 months	39	2.34	0.77	24	2.79	0.69	0.021
	TOMCATS Helplessness 12 months	29	2.40	0.71	26	2.67	0.69	0.167
	TOMCATS Hopelessness 6 months	37	2.18	0.66	24	2.45	0.67	0.124
	TOMCATS Hopelessness 12 months	29	2.08	0.75	25	2.52	0.83	0.046
Health related quality of life								
Li-Tsang, 2008, Musculoskeletal injuries	Short Form Health Survey (SF-36) post-intervention	32	96.79	19.2	31	88.33	19.53	0.02
Sveinsdottir, 2020, Young people not in employment, education, or training	WHODAS 6 months (World Health Organization Disability Assessment Schedule)	37	10.37	9.47	24	9.83	8.64	0.820
	WHODAS 12 months	31	9.70	7.31	25	13.3	8.19	0.088
	CFQ total 6 months (Chalder Fatigue Questionnaire)	38	14.05	6.34	24	14.73	4.74	0.656
	CFQ total 12 months	30	14.19	5.99	26	14.52	5.07	0.823
	SHC total 6 months (subjective health complaints)	36	12.44	10.15	24	18.13	11.58	0.049
	SHC total 12 months	27	14.01	10.35	25	18.05	10.63	0.172
	GWB current 6 months (Global Well-Being)	36	4.56	1.73	22	5.14	2.49	0.344
	GWB current 12 months	30	4.83	1.90	25	4.72	2.13	0.836
	GWB past 6 months	36	3.36	1.74	22	3.91	1.97	0.274
	GWB past 12 months	31	3.65	1.76	25	4.2	2.35	0.317
	GWB future 6 months	35	6.36	2.58	22	6.77	2.56	0.555
	GWB future 12 months	30	8.53	10.87	25	6.06	2.61	0.272
Addiction								
Sveinsdottir, 2020, Young people not in employment, education, or training	AUDIT 6-months (Alcohol Use Disorders Identification Test)	38	3.08	2.55	26	3.62	3.48	0.479
	AUDIT 12-months	31	2.81	2.40	26	3.58	2.93	0.28
	DUDIT 6-months (Drug Use Disorders Identification Test)	37	0.14	0.59	26	0.85	2.17	0.114
	DUDIT 12-months	30	0.27	0.87	24	1.04	2.74	0.194

ITT, intention to treat population; ND, no data; PTSD, posttraumatic stress disorder; SMI, severe mental illness.

* *P*-value as reported verbatim as per study reports (e.g., NS/*P*</*P*=).

Visual inspection of a funnel plot (not shown) may have suggested some asymmetry within an indication of publication bias in smaller trials; however, too few data points existed to draw any firm conclusion. Given we did not meta-analyse, no sensitivity analyses were done.

## Discussion

### Main findings and implications

Ten trials examined the effectiveness of supported employment interventions across populations of people with conditions or circumstances other than serious mental illness alone. Three of the trials were judged to have high RoB. Results suggest that supported employment may be effective outside populations defined by only severe mental illness. Supported employment interventions were more effective than control in returning people to work in trials including people with affective disorders; people with severe mental illness and justice involvement; veterans with PTSD; formerly incarcerated veterans; veterans with spinal cord injury; and in young people not in employment, education, or training. There was an evidence from a trial that supported employment interventions were effective in people receiving methadone treatment (high RoB); although in another trial there was no evidence that supported employment was effective in substance-abuse methadone patients (high RoB). In two other trials, there was no evidence that supported employment interventions were more effective than control in returning people to work in formerly homeless people with mental illness who had recently been housed, and in workers with musculoskeletal injuries (high RoB).

Naturally, we caution that individual trial indications of the effectiveness of supported employment for aiding return to competitive employment and outside populations defined by severe mental illness alone is not an evidence that supported employment will be effective in all populations outside that of severe mental health alone: no general inference should be made to any definable population. The interpretation is rather that supported employment may be of benefit to people with conditions other than severe mental illness alone, or with materially different circumstances to a population of people with severe mental illness alone. Specific population needs should be considered in planning any new trial applying the approaches outside severe mental illness populations.

The included trials have a relatively short follow-up time (mostly 12 months), and it is unclear if benefits to improving employment are likely to be sustained over a longer term. Moreover, it may not always be clear exactly what the principal employment benefits are. Definitions used for competitive employment vary and are not always reported clearly, and where time periods are reported, these range from as little as one day of paid work to four weeks of part-time paid work (*cf*. Appendix Table 2). We recommend future trialists consider defining primary outcomes as *sustained* return to competitive employment within a specified time period, which may better align with real-world aims (Jensen *et al*., 2012). Further, this may allow time needed for secondary impacts on quality of life to be realised (Froud *et al.*, 2020). That said, we note that quality of life was not included in most trials’ measurement of non-vocational outcomes. There is, in general, an absence of evidence on non-vocational measures. Secondary effects might be very important to individuals. For vulnerable groups in particular, being employed might not necessarily indicate improvement in other outcomes and could even result in exacerbation or harm in other domains. We suggest leaders of future trials might consider, measure, and report non-vocational outcomes as secondary outcomes parallel to sustained RTW as the primary. A future Delphi study of core outcome sets may be helpful in this regard. Efforts to collect data that are comparable between trials will facilitate the undertaking and interpretation of systematic reviews and better inform decision-making. Sample sizes were generally small in these studies and thus confidence intervals wide. Even assuming future standardisation of outcome measures, meta-analyses will not excuse small sample sizes: underpinning assumptions regarding the normality of the distribution of underlying effects may not be realistic given population heterogeneity. Mindful of intervention cost, we suggest *a priori* sample sizes should be based on detecting at least medium effect sizes (preferably small-to-medium), or between-group differences in proportion achieving sustained RTW of 20% or less, should be conducted, reported, and targeted in trials. Additionally, we suggest cost-effectiveness analyses should be undertaken. This would improve impact and help policy makers and purchasers make more informed decisions on the basis of better-powered individual trials in the absence of meta-analyses. Assuming control group RTW rate of 16% (i.e., the average across all control groups in this study), a balanced design, and 80% power, then 85 people per trial arm would be needed to detect a difference of 20%; for example, 75% larger than the average sample size of the trials in this review.

Notwithstanding this, the success of supported employment interventions of returning people to work was mirrored in the secondary vocational outcomes that were measured. In studies where supported employment was more effective than TVR in returning people to work, participants earned more and worked more than participants in the control groups.

While the majority of trials in our review identified the tested intervention as IPS, four did not. Two of these were judged high RoB and did not show evidence of an effect (workers with MSK injuries and substance-misuse methadone patients). One was judged low RoB and reported the second largest significant relative increase in return to work (affective disorders) and the other had some concerns identified in the RoB assessment, and showed a significant benefit (formerly incarcerated veterans). Magnitudes of effect varied considerably across trials and the effectiveness of both IPS and non-IPS interventions should continue to be evaluated in populations outside those defined by severe mental illness.

It is not clear why studies in some populations failed to show an effect. Magura *et al.* (2007) speculate that the negative result for competitive employment in customised employment support for substance-misuse methadone users might partly be attributable to unplanned counsellor turnover and the intervention’s reduced potential to provide supported employment at the designed level of intensity. Lones’ substance-misuse pilot study of 45 participants showed a significant effect for IPS at 6 months compared with waiting list control which may be consistent with Magura’s speculation of circumstantial issues effecting outcome (Lones *et al.*).

Heterogeneity, and the small number of studies and participants, negate the testing for any local (e.g., different welfare systems), cultural, or social effects, which might moderate/mediate effects. Such considerations could be set to become more influential factors in the future as the working landscape changes in the wake of COVID-19, and in the establishment of new/increasingly fluid working, cultural, and social norms.

### Comparisons to existing research

Supported employment interventions in populations with severe mental illness alone have been widely researched with several systematic reviews and meta-analyses of IPS-supported employment in a severe mental health population having been published (including three Cochrane reviews) (Kinoshita *et al*., 2013; Suijkerbuijk *et al.*, 2017; Crowther *et al*., 2001) and a narrative review (Marshall *et al.*, 2014). Previous reviews include a review comparing geographical location of population (Modini *et al*., 2016), looking at augmenting interventions alongside IPS (Boycott *et al.*, 2012; Dewa *et al.*, 2017), or assessing effect moderators (Metcalfe *et al*., 2018) all in people with severe mental illness. The present review adds to this body of evidence by identifying which other populations have been sampled in RCTs of supported employment interventions, outside of the traditional client groups of people with severe mental disorders, and whether the interventions are effective in these other populations. Bond *et al*. reviewed and meta-analysed IPS for disorders other than serious mental illness in 2018, concluding that IPS showed some promise. Bond included nine trials with 2902 participants; and five of these are in common with our review. We note that Bejerholm’s trial in people with affective disorder is included as an IPS trial in the Bond review. We identified Bejerholm’s intervention as ‘non-IPS’ as while it was evaluated against the 2008 Supported Employment Fidelity scale (showing good fidelity to this), the intervention was modified by adding two additional principles and renamed individual enabling and support. We excluded four papers included in the Bond review because our focus is on those not in work at the time of study entry. A more recent search and a focus on any type of supported employment intervention resulted in ours including five studies not identified in the Bond review. Unlike the Bond review, we provided details of interventions tested and extracted secondary work and non-work outcomes. We also provide a risk of bias appraisal and chose not to pool very heterogenous data. For these reasons, we believe our review is a comparatively robust appraisal of the evidence.

### Strengths and limitations

We registered this review prospectively in 2017 and followed the PRISMA criteria for reporting in systematic reviews. We searched all relevant bibliographic databases and used backwards citation tracking to identify relevant trials. We only included RCTs in this review and assessed trials for methodological quality with the Cochrane Risk of Bias Tool v2.0. We did not contact experts and excluded non-English language studies and cannot exclude the possibility that other trials exist. However, any trials missed for this reason are likely to be smaller, lower quality trials. Given this, and the already heterogeneous nature of the included studies, small missed trials would be unlikely to change our overall conclusion.

It is likely that previous trials, targeting people with severe mental illness, included individuals from some of the populations tested in the studies were included in this review. This review extends knowledge by exploring whether these individually more homogenous groups benefitted from supported employment approaches. Although one principle of supported employment in general, and IPS specifically, is non-exclusion, in reality many trials assessing IPS were conducted in a population of people with severe mental illness and specifically excluded complicating factors like homelessness and justice involvement. We did not pool trials due to the obvious population heterogeneity. We presented descriptive statistics alone and no inference should be made to a wider definable population.

Supported employment interventions tested within RCTs inevitably differ from real-life settings; for example, unlimited support can only be provided for the duration of the study follow-up. Thus, although the findings of this review are promising, phase IV trials and audits of existing programmes may be beneficial. More research is needed to identify predictors of outcome and to establish content and delivery factors contributing to success in populations outside the severe mental illness populations.

## Conclusions

Supported employment interventions may be useful for people with severe mental illness and some added complications, as well as for some non-severe mental illness groups, including people with affective disorders, mental health and justice involvement, veterans with PTSD, spinal injury, or formerly incarcerated veterans, people receiving methadone treatment, and young people not in employment, education, or training. Defining competitive employment and increasing (and standardising) measurement of non-vocational outcomes, such as quality of life, may help to improve research in this area.

## Footnotes

† The authors state RTW outcomes were collected three weeks after the programme and note the four week 18-hour outcome is defined by the Employment Ordinance of Hong Kong. We presume therefore that employment could be started during the programme so the outcome could be achieved.

## Supplementary material

For supplementary material accompanying this paper visit http://dx.doi.org/10.1017/S1463423621000827. click here to view supplementary material
