# Supplementary material for: The effects of supported employment interventions in populations of people with conditions other than severe mental health: a systematic review
Source: Prim Health Care Res Dev. 2021 Dec 9;22:e79. doi: 10.1017/S1463423621000827 (PMC8724223; doi:10.1017/S1463423621000827)
Supplement: Supplementary file 1 [file phcsup.zip › S1463423621000827sup001.pdf]

Appendix (1) to '*The effects of supported employment interventions in populations of people with conditions other than severe mental health: A systematic review*' – Probyn et al.

Appendix 1 Table 1 – Additional details of included study interventions (see below for a list of abbreviations)

Study ID	Intervention condition	Control condition
Bejerholm 2017	Assessed the Individual Enabling and Support (IES) intervention for people with affective disorders. The intervention is based on IPS with the inclusion of two additional principles added to the eight IPS principles.<br>An employment specialist works closely with the participant, outpatient team, family, Social Insurance Agency (SIA), Public Employment Service (PES), and employers guide IES. The professional role requires high quality, empathetic counselling corresponding to the ten IES principles 1) handling change and developing motivational and cognitive strategies, 2) having a time-use pattern that supports work-life balance, 3) integration of IES with mental health treatment, 4) competitive employment as a primary goal, 5) eligibility based on client choice, 6) rapid job search, 7) job search based on personal preferences, 8) on-going support and work accommodations as needed, 9) benefit counselling at an early stage, and 10) systematic recruitment and quality engagement with employers. Principles 3–10 correspond to the IPS model. The extent of the counselling is	TVR was delivered by various professionals in several settings and organisations, *e.g.* healthcare, municipality, SIA and PES. TVR is regulated by the social benefit and unemployment security system. The service is customised to a lesser extent; to a larger extent it is facilitated in several assessment stages that are regulated by organisations. The first step involves reducing symptoms and increasing work ability at a mental health service (1h per week). Step 2 involves assessment of 50% work capacity (10–20 h per week). If work capacity is not met, the participant is encouraged to enter Step 3 with pre-vocational activities at the municipality (5–20 h per week). The last step is vocational training during internship placements (20–40 h per week), and these can lead to employment positions.

	customised to fit the intervention need of the participant.<br>• Caseload: ~20<br>• Two employment specialists in the study<br>• Good fidelity reported to supported employment on the Becker 2008 Supported Employment Fidelity scale	
Bond 2015	Assessed IPS for people with severe mental illness with justice involvement where IPS employment specialists received a day's training on criminal justice issues. The IPS condition followed the principles of IPS supported employment, enhanced with a daylong training for IPS employment specialists on criminal justice issues. The employment specialists on the two IPS teams involved in the study received on-going consultation, including twice-monthly conference calls from a senior IPS trainer throughout the duration of the study.<br>• Caseload: 20<br>• Two IPS treatment teams<br>• Good fidelity to IPS reported on the supported employment fidelity scale	Work Choice was based on the empirically validated job club model tailored for persons with psychiatric disabilities. It facilitated a self-directed job search, helping clients with résumé preparation, interview skills, and job leads. Classes were scheduled weekly at two conveniently located sites. The curriculum included training in application procedures, job search strategies, and linkage services. The classes were held in a room with computer workstations for applying online for jobs. The Work Choice staff helped participants find job openings and navigate the online application process. Two half-time workers with lived experience of mental illness staffed the programme.
Davis 2012	Assessed IPS delivered to veterans with PTSD, where the employment specialist was integrated into the clinical mental health or PTSD treatment team. The IPS specialist was integrated into the clinical mental health or PTSD treatment team and carried out all phases of the vocational services, and provided predominantly community-based	VRP followed the standard care in place at Tuscaloosa Medical Center, which included one or more of these components:<br>Routine prevocational testing and evaluation for all patients on referral to VRP, vocational rehabilitation therapy that provided a work regimen with monetary incentives derived from contracts whereby

	services, assertive engagement and outreach in community-based, and provided continuous, time-unlimited, follow-along supports for vocational services. IPS involved rapid job search and customised placement in diverse competitive jobs, with ongoing work-based vocational assessment and assistance in finding subsequent jobs, if needed.<br>Group supervision was regularly scheduled with IPS specialists and an IPS supervisor.<br>• Caseload: up to 25 clients<br>• Fair fidelity to IPS reported on the supported employment fidelity scale	participants are paid on a piece-rate basis related to their production, and a transitional work programme that included a temporary work experience either within Tuscaloosa Medical Center or in community settings (called the transitional work external or transitional work experience). The VRP model featured a VRP specialist who carried out some, but not all, phases of the vocational service.
Le Page 2016	Assessed IPS principles in a vocational rehabilitation programme (*'About Face')* for formerly incarcerated veterans. About Face was a one-week standardised vocational rehabilitation group-based training in which participants begin to develop a list of employment experiences, aspirations, and skills. Two supported employment specialists provided the intervention. Both were rehabilitation councillors trained either through formal coursework and practical experience or through attending specialised training. A clinical psychologist with experience in overseeing IPS programs supervised the rehabilitation councillors. Veterans assigned to AF+IPS received the customised group and additional services based on the IPS model of supported employment. IPS was provided by one of two supported employment specialists (SESs). Both SESs were rehabilitation	A one-week customised vocational rehabilitation program within About Face, which was a group–based programme that has been successful in assisting the population with finding employment. The group typically includes three to seven participants. Within the group, veterans begin by developing a list of employment experiences, aspirations and skills. Veterans develop a basic but professional résumé simple enough to prepare with word processing software. A large section of the group focuses on the specific problems often encountered by veterans with felony histories, with examples and rationales to help develop personalised responses. Veterans receiving AF received only the group-based programme.

	counsellors trained either through formal coursework and practical experience or through attending customised training. A clinical psychologist with experience overseeing IPS programmes supervised both.<br>Although the IPS model served as the basis for the intervention in the AF+IPS condition, several deviations were incorporated, and as such, the programme used was not viewed as meeting the standards of evidence-based supported employment). All veterans were required to participate in the vocational classes immediately after enrolment. Although classes were required, all veterans in AF+IPS began the job search process within two weeks. In contrast to standard IPS, in AF+IPS, higher caseloads of up to 35 per SES were allowed due to the lower incidence of serious mental illness compared with typical populations for IPS.<br>• Caseload: up to 35<br>• Non-IPS supported employment; reported as not classed as IPS on fidelity scale	
Lones 2017	Assessed IPS for patients with opioid use disorders enrolled in an opioid treatment (methadone maintenance therapy) programme, within and outside of a clinical setting. While the IPS intervention was conducted in the treatment site's clinic, the IPS specialist should spend 60% of their time outside of the clinic setting. An outside trainer supervised the IPS specialist. The same specialist	Waiting list for IPS for six-months, after six-months participants on the waiting list became also eligible for IPS.

	provided all IPS participant services throughout the trial.<br>• Caseload: 22 (first six months)<br>• The study included only one treatment agency and one IPS specialist.<br>• Fair fidelity to IPS reported on supported employment fidelity scale	
Li-Tsang 2008	Assessed 'The Job Placement and Support Program for Injured Workers', which involved an individual interview, vocational counselling, and on-going support once a job was secured. The programme entailed individual interviews, vocational counselling, job preparation, and job seeking and it lasted for three weeks. Five case managers, who had previous training on vocational counselling and job placement, were involved in the training programme.<br>The case manager conducted an initial interview, then set up a personal data base for each subject which included the personal information, injury and work issues, medical history and previous work history, assessment records on functional and work capacities, other job skills, academic background, and job preferences. The case manager helped the subject formulate his/her concrete action plan on job placement, set realistic a job goal matching with personal interests and current functional status or work capacities. Subjects were also assisted to identify 'job barriers' and to build realistic goals about job expectations.<br>• Caseload not reported	Subjects in the self-placement group first met with a social worker at the Workers' Health Centre and then advised to search for jobs from the websites of the Labour Department or through the newspaper. This is the routine referral for workers who are seeking for jobs; in this study, these subjects who received the provision of services at the Workers Health Centre were regarded as the self-placement group.

	• Non IPS supported employment, not reported as assessed on fidelity scale	
Magura 2007	Assessed the 'Customized Employment Supports (CES) Model' for substance misuse methadone patients. The intervention involved a counsellor working intensively with a small caseload of substance misuse methadone patients to overcome vocational and non-vocational 'barriers' to employment with the goal of rapid placement in competitive employment. The intervention emphasised intensive individual counselling, rapid job search, and therapeutic fieldwork. CES counsellors worked with patients to customise non-vocational barriers while helping them seek employment in a job of their choice. One important engagement strategy consisted of fieldwork; that is, activities in which counsellor and patient interact in the community rather than in the clinic. Vocationally relevant learning activities that take place on 'neutral turf' help to customise the counsellor–patient relationship and accelerate the development of patient trust and openness. The resulting customised and intense interventions limited each CES counsellor's caseload to approximately 15 'active' patients at a time. CES counsellors met face-to-face with patients up to three times per week and often had further contact by phone or e-mail. As noted, some of the face-to-face contacts occurred in the community rather than in the office.<br>• Case load: 15	The existing services at each site offered non-intensive, individual vocational counselling as well as the opportunity for participation in vocationally oriented groups. The counselling activities were either prevocational (*e.g.*, working with patients on hygiene issues; insuring that patients had appropriate documentation) or job seeking (*e.g.*, helping patients write a résumé), depending upon the needs and desires of the patients. Virtually all contacts with counsellors occurred in the clinic. Some patients, however, were referred to independent job training and job placement agencies. Paralleling the CES staffing, there was one counsellor offering standard vocational services at each site. These counsellors, who also provided the same standard services to other methadone patients not in the study.

	• Non-IPS supported employment, not reported as assessed on fidelity scale	
Ottomanelli 2012, 2014	Assessed the 'Spinal Cord Injury Vocational Integration Program (SCI-VIP)' for veterans with spinal cord injury. The intervention followed IPS and the principles of integrated treatment, rapid engagement, competitive employment, belief success is possible regardless of severity or type of disability, on-going support, veteran preferences, community-based services and personalised benefits counselling.<br>The authors sought to follow the evidence-based principles of IPS as closely as possible including integrated vocational and medical rehabilitation treatment, rapid engagement in job finding, competitive employment, inclusion regardless of severity or type of disability, on-going job support, and focus on participant preferences. Services were primarily provided in the community, rather than in office or hospital settings, and access to personalised benefits counselling was included. The provision of these services was by a VR counsellor who was hired for the study, trained in the IPS SE model, and integrated as a provider among the SCI interdisciplinary care team in the SCI centre.<br>• Caseload not reported<br>• Fair fidelity to IPS reported on supported employment fidelity scale	The treatment-as-usual (TAU) condition typically involved referrals to VR services outside the Veterans' Affairs SCI center. In this condition, there was not a single provider of VR services who was part of the SCI interdisciplinary care team at the SCI centre. Rather, subjects in the TAU condition were referred by the research coordinator back to their clinical SCI interdisciplinary team members (*e.g.*, physiatrist, psychologist, social worker) who would provide them with a referral to an agency or provider who was not part of the SCI centre and these subjects may or may not have received any additional VR services.
Poremski 2015	Assessed IPS for mental illness in formerly homeless recently housed people. In this	Participants customised to the control condition were free to seek employment by any means of their

	intervention employment specialists were trained and supervised by a senior member of an experienced local IPS service. They worked closely with the clinical teams from whose caseloads their clients were drawn.<br>• Caseload not reported<br>• Fair-to-good fidelity to IPS reported on supported employment fidelity scale	choice, with some support from their case managers. Available services included training with eventual placement in jobs reserved for people receiving basic social assistance or disability payments. Community-based services for people who were homeless were also available. None of these services were integrated into the clinical teams or offered continuous time-unlimited personalised support.
Sveinsdottir 2020	Assessed IPS for young people not in employment, education, or training. An IPS team leader supervised the job specialists, and an external IPS trainer advised the team. Because the study population had various social and health related challenges that did not necessarily involve mental illness, the IPS principle of integrating services with mental health treatment was not implemented, although job specialists contacted health personnel involved in the treatment of individual participants in cases where this was applicable and accepted by the participant. The<br>intervention was offered for up to three years, and the duration and intensity depended on individual needs and preferences.<br>• Caseload not reported<br>• Fair fidelity to IPS reported on supported employment fidelity scale in three out of five fidelity reviews	Traditional vocational rehabilitation (TVR) intervention represented treatment as usual and served as an active control condition. The traineeships involved testing work capability and providing preparatory work training adapted to individuals' challenges and skill levels, in a sheltered setting with close follow-up. According to usual practice, participants were allocated to various sheltered businesses in the area providing different types of work settings, including food and catering, child care, mechanic services, transportation services, and warehouse handling, based on individual interests and goals as well as availability. The intervention was offered for up to two years, and the duration was customised to the individual's options on the labour market. The usual intensity of the intervention is full-time, with a requirement of ≥50% of full-time.

Int.= intervention, contr.= control

List of abbreviations

AF	About Face
C-STAI	State Trait Anxiety
CES	Customised Employment Support
IES	Individual Enabling and Support
IPS	Individual Placement and Support
ITT	Intention to treat
PES	Public Employment Service
PTSD	Posttraumatic stress disorder
RCT	Randomised controlled trial
RR	Risk Ratio
RTW	Return to work
SCI	Spinal Cord Injury
SCI – VIP	Spinal Cord Injury Vocational Integration Program
SD	Standard Deviation
SES	Supported employment specialist
SF 36	Short Form Health Survey
SIA	Social Insurance Agency
SMI	Severe mental illness
SP	Self-placement
SS	Standard services
TAU	Treatment as usual
TVR	Traditional vocational rehabilitation
VA	Veterans Affairs
VRP	Veterans' Health Administration Vocational Rehabilitation Program

Appendix Table 2 – Definitions of competitive employment

Lead author, year	Definition used for competitive employment
Bejerholm, 2017	NR ("competitive employment as a primary goal")
Bond, 2015	"Jobs in integrated work settings in the competitive job market at prevailing wages, with supervision provided by personnel employed by the business."
Davis, 2012	"A job for regular wages in a setting that was not set aside, sheltered, or enclaved, that is, the same job could be held by people without a mental illness or disability and was not a set-aside job in the VRP [Vocational Rehabilitation Programme]. Day labor (that is, pick-up cash-based day jobs for yard work, babysitting, manual labor, and so forth) and military drill were not counted as competitive employment; however, data on income from all sources were collected."
LePage, 2016	NR. ([competitive employment] "was confirmed by either an SES's [supported employment specialists] or study coordinator's review of paystubs. Community visits, contacts with employers, or other means.")
Lones, 2017	"Jobs in integrated work settings in the competitive job market at prevailing wages with supervision provided by personnel employed by the business"
Li-Tsang, 2008	Four-weeks of work or more for at least 18 working hours per week.
Magura, 2007	"Competitive paid employment, full- or part-time, at some time during both the first 6- month follow-up

	period and the 7- to 12-month follow-up period. This employment may have been the same job that extended over the two follow-up periods or two (or more) different jobs during the two follow-up periods. (However, the patient need not have been competitively employed for the entire 12 months.) This summary measure was selected because it combines both attaining competitive employment and continuity of competitive employment activity over the follow-up period."
Ottomanelli, 2012	"The primary endpoint was competitive employment, meaning a paying job earning at least minimum wage in the community. Volunteer work and sheltered employment did not qualify as employment for the purposes of the present study."
Ottomanelli, 2014	"Having a paying job in the community earning minimum wage or higher."
Poremski, 2017	"Whether or not the participant had worked at least one day in a 30-day period."
Sveinsdottir, 2020	Paid employment in the competitive labour market, of at least one day, and excluding subsidised or unpaid work.

NR=Not reported

Appendix 1 Note 1 – Search String used for Pubmed

((("Employment, Supported"[Mesh]) OR ("supported employment" OR "individual placement support" OR "individual* placement and support"))) AND ((((((quasi OR RCT OR "random*" OR "non-random*" OR "nonrandom*" OR "non-RCT" OR "non RCT" OR "clinical trial*" OR "evaluation stud*")) OR "Random Allocation"[Mesh]) OR ("Non-Randomized Controlled Trials as Topic"[Mesh] OR "Randomized Controlled Trial"[Publication Type] OR "Randomized Controlled Trials as Topic"[Mesh])) OR ("Clinical Trial"[Publication Type] OR "Clinical Trials as Topic"[Mesh] OR "Controlled Clinical Trial"[Publication Type] OR "Evaluation Studies"[Publication Type])) OR ("Evaluation Studies as Topic"[Mesh] OR "Evaluation Studies"[Publication Type]))*
